# Nanoscale organization of cardiac calcium channels is dependent on thyroid hormone status

**DOI:** 10.1152/ajpheart.00272.2024

**Published:** 2024-10-04

**Authors:** Amanda Charest, Nicholas Nasta, Sumaiyah Siddiqui, Silvia Menkes, Anvin Thomas, Dana Saad, Jake Forman, Xueqi Huang, Cristina P. Sison, A. Martin Gerdes, Randy F. Stout, Kaie Ojamaa

**Affiliations:** ^1^Department of Biomedical Sciences, New York Institute of Technology College of Osteopathic Medicine, Old Westbury, New York, United States; ^2^Biostatistics Unit, Office of Academic Affairs, Northwell Health, New Hyde Park, New York, United States

**Keywords:** junctophilin-2, L-type calcium channel, ryanodine receptor-2, STORM, thyroid

## Abstract

Thyroid hormone dysfunction is frequently observed in patients with chronic illnesses including heart failure, which increases the risk of adverse events. This study examined the effects of thyroid hormones (THs) on cardiac transverse-tubule (TT) integrity, Ca^2+^ sparks, and nanoscale organization of ion channels in excitation-contraction (EC) coupling, including L-type calcium channel (Ca_V_1.2), ryanodine receptor type 2 (RyR2), and junctophilin-2 (Jph2). TH deficiency was established in adult female rats by propyl-thiouracil (PTU) ingestion for 8 wk; followed by randomization to continued PTU without or with oral triiodo-l-thyronine (T3; 10 µg/kg/day) for an additional 2 wk (PTU + T3). Confocal microscopy of isolated cardiomyocytes (CMs) showed significant misalignment of TTs and increased Ca^2+^ sparks in thyroid-deficient CMs. Density-based spatial clustering of applications with noise (DBSCAN) analysis of stochastic optical reconstruction microscopy (STORM) images showed decreased (*P* < 0.0001) RyR2 cluster number per cell area in PTU CMs compared with euthyroid (EU) control myocytes, and this was normalized by T3 treatment. Ca_V_1.2 channels and Jph2 localized within a 210 nm radius of the RyR2 clusters were significantly reduced in PTU myocytes, and these values were increased with T3 treatment. A significant percentage of the RyR2 clusters in the PTU myocytes had neither Ca_V_1.2 nor Jph2, suggesting fewer functional clusters in EC coupling. Nearest neighbor distances between RyR2 clusters were greater (*P* < 0.001) in PTU cells compared with EU- and T3-treated CMs that correspond to disarray of TTs at the sarcomere *z*-discs. These results support a regulatory role of T3 in the nanoscale organization of RyR2 clusters and colocalization of Ca_V_1.2 and Jph2 in optimizing EC coupling.

**NEW & NOTEWORTHY** Thyroid hormone (TH) dysfunction exacerbates preexisting heart conditions leading to an increased risk of premature morbidity/mortality. Triiodo-l-thyronine (T3) optimizes cardiac excitation-contraction (EC) coupling by maintaining myocardial T-tubule (TT) structures and organization of calcium ion channels. Single-molecule localization microscopy shows T3 effects on the clustering of ryanodine receptors (RyR2) with colocalization of L-type calcium channels (Ca_V_1.2) and junctophilin-2 (Jph2) at TT-SR structures. Heart disease with subclinical hypothyroidism/low T3 syndrome may benefit from TH treatment.

## INTRODUCTION

It has long been recognized that thyroid dysfunction has profound effects on cardiac and circulatory function (1–3). Both excess and deficiency of thyroid hormones (THs) can exacerbate preexisting heart conditions with coronary artery obstruction, contractile dysfunction, and atrial or ventricular fibrillation, thereby contributing to a higher risk of premature morbidity and death (4–6). Recent observational studies further support increased cardiovascular risk associated with more severe symptoms and poor prognosis in patients with subclinical hypothyroidism (7–11). Clinical trials restoring thyroid hormone function in patients with heart failure (HF) have shown significant improvements in ventricular remodeling and contractile function (12–15). Importantly, an extensive body of basic cellular- and molecular-focused research has provided the rationale supporting the beneficial effects of restoring normal thyroid function in cardiovascular disease (16–19).

In our recently published study focused on normalizing thyroid function in an animal model of heart failure, we observed a restoration of T-tubule (TT) integrity with subsequent normalization of Ca^2+^ transients and improved contractile function (20). Results from numerous studies have convincingly shown that adverse remodeling of TT structures with alterations in dyad structures occurs in HF in both animal models and in human disease (21–24) (reviewed in Ref. 25). With these structural changes, the juxtaposition of L-type Ca^2+^ channels (LTCCs) with ryanodine receptors (RyR2) at junctional sarcoplasmic reticulum (jSR) is disrupted resulting in loss of synchrony of Ca^2+^-induced Ca^2+^ release (CICR) and altered initiation of excitation-contraction (EC) coupling. Therefore, the clustering of dyadic ion channels and colocalization of associated proteins including junctophilin-2 (Jph2) are critical determinants of effective cardiomyocyte contractile activity (26–28).

Many of the physical, phenotypic, and functional characteristics of the failing heart are observed in the hearts of patients with hypothyroidism and have been referred to as the “fetal” phenotype (reviewed in Refs. 1, 3, and 29). Our recent RNA sequencing studies that focused on the expression of RyR2, LTCC, bridging integrator1 (BIN1), and Jph2 in failing hearts and hypothyroid hearts indicated that Jph2 is triiodo-l-thyronine (T3) responsive and may be a key determinant of dyad organization (20, 30). Other investigations using induced pluripotent stem cell (iPSC)-derived cardiomyocytes and engineered heart tissues have documented a regulatory role of thyroid hormones on T-tubule development and maturation, and promotion of ion channel organization at the dyad (31–33). Thus, we undertook the present study to investigate the effects of thyroid hormones on RyR2 cluster formation, and colocalization of LTCC and Jph2 within clusters that determine efficient EC coupling in the heart.

## MATERIALS AND METHODS

### Animal Model and Treatment Protocols

The Institutional Animal Care and Use Committee of the New York Institute of Technology College of Osteopathic Medicine approved the study protocols. All animals were treated in accordance with the National Institutes of Health’s *Guidelines for the Use and Care of Laboratory Animals* (HHS Pub. No. 85-23). As we have previously published (30), thyroid hormone deficiency was induced in female Sprague–Dawley rats (220–250 g; Envigo RMS, Indianapolis, IN) by ingestion of 6-propyl-2-thiouracil (0.025% PTU; Sigma-Aldrich, St. Louis, MO) dissolved in drinking water provided at ad libitum for 8 wk. Rats were then randomized to continued PTU treatment (PTU group) or triiodo-l-thyronine (T3; Sigma-Aldrich; St. Louis, MO) treatment in PTU-containing drinking water for an additional 2 wk (PTU + T3 group). Stock T3 preparation and dilution in drinking water was calculated to deliver 10 µg/kg/day based on body weight and daily water consumption as previously published (20, 34). Control rats (euthyroid, EU group) consumed untreated water. Rats were housed under controlled temperature conditions, 12-h:12-h light/dark cycles, with standard rat chow and water available ad libitum. Animal numbers per treatment group for each analysis are indicated in the text.

### Blood Thyroid Hormone Analysis

Following the 10-wk treatment period, animals were anesthetized, and a left thoracotomy exposed the heart. Blood was collected from the right ventricle in the anesthetized animals at approximately the same time of day. Blood plasma was separated, aliquoted, and stored at −20°C until enzyme immunoassay of total T3 and total T4 (Monobind, Lake Forest, CA).

### Ventricular Myocyte Isolation

Left ventricular myocytes were enzymatically isolated as we have previously described in detail (30). The isolated cardiomyocytes were plated onto laminin-coated eight-well glass-bottom chamber slides (Ibidi) in medium M199 containing 10% FBS and 2,3-butanedione monoxime (BDM, 10 mM) and allowed to adhere for 2 h before processing for T-tubule and calcium-spark analyses or fixation and antibody staining for two-dimensional (2-D) and three-dimensional (3-D) stochastic optical reconstruction microscopy (STORM) imaging.

### Confocal Imaging of T-Tubule Organization

As we have previously published (20), adherent live cardiomyocytes were stained with di-8-ANEPPS (AminoNaphthylEthenylPyridinium) dye (5 µM in HBSS with 10 mM BDM; Biotium, Fremont, CA) for 20 min at 37°C. Live cells were imaged using the Axio Observer.Z1/7 (Zeiss 980 LSM) microscope with Plan-Apochromat 63×/1.40 Oil DIC M27 objective. Pinhole was set to 1.00 AU/59 µm, pixel time was 0.51 µs, imaging device was MA-Pmt1, detector type was Multialkali-PMT, detector with gain at 650 V. Nine slices at 3.2-µm intervals were captured, scaling per pixel was 0.132 µm × 0.132 µm × 0.400 µm, image size was 1,024 × 1,024 pixels scaled to 134.69 µm × 134.69 µm. The bit depth was 8. The transverse-oriented elements (TEs) of the T-tubule system of each image were analyzed for density and regularity, and the index of TE-tubule integrity (%TT_int_ = TE density × TE regularity) was computed using AutoTT, an automated analysis program developed by Guo and Song (35).

### Calcium Spark Analysis

Adherent live cardiomyocytes in eight-well chamber slides were loaded with 5 µM Fluo-4-AM (Invitrogen) in normal Tyrode’s solution, consisting of (in mM) 140 NaCl, 4 KCl, 10 glucose, 10 HEPES, 1 MgCl_2_, and 1.8 CaCl_2_ (pH 7.4), containing 16 µM Pluronic F127 (Invitrogen). Following incubation for 20 min at 37°C, cells were washed in Tyrode’s solution to allow de-esterification. Cardiomyocytes were not paced before image capture. Myocytes in which calcium waves were visibly evident and, therefore, contracting spontaneously due to membrane depolarization and possible loss of membrane integrity were not selected for imaging. Spontaneous Ca^2+^ sparks or “leaks” from the SR were recorded from 10 randomly selected cardiomyocytes per chamber using the Zeiss 980 microscope (housed in a 37°C heated chamber) with the LD LCI Plan Apochromat 40×/1.2 Imm Korr DIC M27 water-immersion objective, pinhole 1.20 AU/45 µm, zoom of 3.0, scan mode was in line scan, with a scan zoom of 3.0, pixel time 1.33 µs, and scan speed 7 unidirectional. Spectral GaAsP-PMT detector with gain set at 818 V. Images of calcium sparks were acquired with the scanning line (70 µm length) placed parallel to the longitudinal axis of the myocyte at approximately the *z*-axis center and at a scanning speed of 406 lines/s for 1,800 frames (pixel dimension = 0.089 µm × 0.089 µm). Line-scan images of 10 cells per heart were saved to the Zen 3.0 program and converted to 8-bit grayscale before analysis using the SparkMaster plugin in ImageJ analysis software (36).

### Immunofluorescence Staining for 2-D and 3-D STORM

Ventricular myocytes isolated on the same day from different study groups were plated into separate wells on laminin-coated eight-well chamber slides. For immunostaining, adherent cardiomyocytes were fixed in 4% paraformaldehyde (PFA) for 20 min, then washed with PBS, and permeabilized and blocked with 0.2% Triton X-100 plus 5% goat serum in PBS for 1 h. Myocytes were incubated overnight at 4°C with mouse monoclonal anti-RyR2 (DyLight 550 conjugated) antibodies (1:200; NBP2-80143R/C3-33; Novus Biologicals, Centennial, CO) and rabbit polyclonal anti-Jph2 (1:400; Invitrogen 405300, Thermo Fisher Scientific) or rabbit polyclonal anti-Ca_V_1.2 (1:400; ACC-003; Alomone, Jerusalem, Israel) antibodies. Immunoblot analysis of rat myocardial samples confirmed that Invitrogen’s Jph2 antibody recognized the full-length protein preferentially. Following PBS wash to remove unbound primary antibodies, cells were incubated with secondary antibodies of highly cross-absorbed goat anti-rabbit IgG(H + L) Alexa Fluor Plus-647 (1:2,000; A21245, Invitrogen, Thermo Fisher Scientific) for 1 h at room temperature to label either Jph2- or Ca_V_1.2-bound primary antibodies. Immunolabeled cells were briefly fixed with 4% PFA and then washed and stored in PBS containing sodium azide at 4°C until they were imaged using the Nanoimager-S superresolution microscope [Oxford Nanoimaging (ONI), Oxford, UK] equipped with an Olympus 1.4 NA 100× oil immersion super-apochromatic objective. Color channel mapping was calibrated separately for 2-D and 3-D image capture at the start of each STORM imaging session using 0.1-µm 200-nm-diameter Tetraspek beads (Thermo Fisher Scientific). For 2-D calibration, *x*- and *y*-coordinates of 10,000 Tetraspek beads in several fields of view were tracked. For 3-D calibration, a cylindrical lens was placed in the light path to create astigmatic distortion of the Gaussian spots that depend on the distance from the focal plane of each bead along the *z*-axis to create a three-dimensional map covering the field of view. As we have previously described (30), cells were incubated in a blinking induction buffer (BCubed buffer; ONI) during STORM imaging with a fresh buffer change every 30–40 min. The optics of the Nanoimager S separates light of wavelengths longer than 640 nm from all shorter wavelengths by directing it to different halves of the sCMOS Hamamatsu orca flash 4 V3 camera chip. Single-molecule localization data from the excitation of each fluorophore were recorded sequentially, first at 640 nm, then at 561-nm wavelength lasers to avoid cross-excitation or bleed through between channels. Each image was acquired as a series of 5,000 raw frames at 50 ms/frame at each wavelength. The focal plane used for image capture was taken as the brightest fluorescence signal in the 561-nm channel determined by visual inspection, indicating internally located RyR2s.

### STORM Image Analysis and Data Computation

Raw image data were corrected for drift and filtered according to a standard protocol integrated into ONI imaging software that included applying a *P* value-based cutoff for each localization through calculation of the χ^2^ value of the area under the curve of the Gaussian signal detected on the camera for each localization (37). A *P* value of 0.25–1.0 was applied to the 647-nm channel localizations to remove data points of poor resolution (i.e., background noise). Subsequently, these filtered image data were exported from the Nanoimager NimOS software v.1.19 and loaded into the LumeVR software program for localization microscopy data analysis developed uniquely for this study by LumeVR Inc [Oxford, UK; Python script available upon request (38)]. The daily 2-D channel mapping calibration was used to obtain subnanometer pixel mapping for the simultaneous two-channel image capture that is built into the Nanoimager. The 3-D calibration mapping of X-Y Gaussian distortion that was established at the beginning of each imaging session was used to calculate *z*-coordinates for images obtained in 3-D mode with the cylindrical lens employed. For each resulting 2-D SMLM image, two identical regions of interest (ROI) were delineated based on area for 2-D images (3.0 × 10^8^ nm^2^) that were away from the cell periphery and from areas visually identified as occupied by nuclei. For each ROI, cluster analysis of data captured individually in each channel was accomplished by density-based spatial clustering of applications with noise (DBSCAN) as described by Ester et al. (39). Specific parameters used to define RyR2 clusters were based on our prior study (30), and on other investigator-established measurements of RyR2 clusters (40–42) as follows: minimum number of localizations set to 10, and the minimum and maximum distances between the furthest two points within a cluster were set to 50 and 800 nm, respectively. The LumeVR analysis program counted the number of localizations in the 561-nm channel within each defined RyR2 cluster. To determine the Jph2 localizations associated with each RyR2 cluster, a circular area with a radius of 210 nm around the RyR2 cluster center point was used to count the number of localizations in the 647-nm excitation laser channel corresponding to Jph2. Nearest neighbor distances (NNDs) between RyR2 clusters were measured from the centroid of each cluster. The resulting datasets from each ROI were exported to spreadsheets for calculations of means and medians of RyR2 and Jph2 localizations per cluster, Jph2/RyR2 ratios, nearest neighbor distances (NNDs) and percent RyR2 clusters without Jph2 localizations. Results from the two ROIs per cell were averaged, with results from 12 to 15 cells per heart expressed as a mean value per animal or plotted as individual cell data from five to six hearts per study group. In addition, RyR2 cluster sizes were separated into bins of 25 localizations from 0 to 400. Histograms were derived from the calculated average of Jph2 localizations or NND values for each bin size.

Similar to 2-D image processing, the raw 3-D image data were filtered and exported from NimOS to the LumeVR analysis platform with three-axis values per point. For each cell image, two identical ROIs were delineated based on volume (3.0 × 10^11^ nm^3^). DBSCAN was used to identify RyR2 clusters, and the minimum localizations that defined a cluster were set to 10, with the maximum localization number set to 1,000. In a subset of DBSCAN analysis of RyR2 clusters, the minimum localization of clusters was set to 4, and clusters with 4–9 localizations were analyzed for comparison to clusters of ≥10 localizations. The RyR2 cluster size limits were set to a minimum of 50 nm and a maximum of 800 nm. The number of localizations of Ca_V_1.2 and Jph2 within a sphere of 210 nm radius from the centroid of each RyR2 cluster were recorded. Similar to 2-D image analysis, measured end points were RyR2 cluster size (furthest distance between any two localizations), cluster volume, number of RyR2 localizations per cluster, and number of localizations corresponding to Ca_V_1.2 and Jph2 within a sphere of 210 nm radius of the RyR2 cluster centroid. Calculated values from two ROIs per cell were averaged, and a mean value was obtained for each parameter from all images (12–15 cells) per heart. Additional analysis involved statistical comparison among the study groups using individual cell data from each heart.

### Statistical Analyses

Data presentation and analysis are detailed in the table and figure legends. Data were assessed for normality distribution and equal variance, and statistical significance among groups was determined by one-way ANOVA, and post hoc pairwise comparisons of group means used Tukey’s multiple comparisons test (Prism software v. 10; GraphPad Prism Software, San Diego, CA). Data are plotted as means ± SD of individual cell data from the five or six hearts per group or as violin plots showing median and quartiles. Statistical significance was set at *P* < 0.05.

Some data were plotted as histograms with cluster sizes segregated into bins. Due to the hierarchical nature of the binned data, a linear mixed-effects (LMEs) model was used to assess the relationship between group (EU, PTU, PTU + T3) and each outcome (frequency of RyR2 clusters, Jph2 loc/RyR2 cluster, and NND of RyR2 clusters). The model included fixed effects of group and RyR2 Loc Bin size, and random effects for animals and cells. All three outcomes were log transformed to meet the model assumptions. Post hoc pairwise comparisons were performed and adjusted using Tukey’s honestly significant difference. The level of significance was set at 5% unless stated otherwise. These analyses used SAS software v.9.4 (Cary, NC).

## RESULTS

### Animal Model Physiological Data

**Figure 1.**
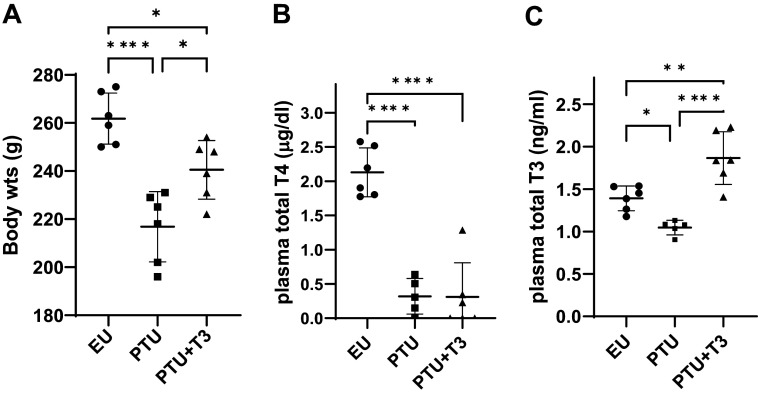
Animal model physiological parameters. *A–C*: body weights (in g) of animals in each experimental group (euthyroid, EU; hypothyroid, PTU; PTU plus T3 treated, PTU + T3) at the study terminus (*A*), plasma total 3,5,3′,5′-tetraiodo-l-thyronine or thyroxine (T4) (*B*), and 3,3′,5-triiodo-l-thyronine (T3) (*C*) measured at the end of the study. Data are means ± SD, dots are individual animal values; 5 to 6 animals/group. Statistical analysis used one-way ANOVA, post hoc Tukey’s multiple comparisons test of group means. Significance between groups is indicated by brackets; *P < 0.05, **P < 0.01, and ****P < 0.0001.

Thyroid hormone deficiency resulted in a significant reduction in animal body weights within the 10-wk PTU treatment period (Fig. 1*A*). T3 treatment of the hypothyroid PTU-treated animals increased their body weights, but normalization was not attained within this 2-wk period since numerous physiological processes determine body weight. PTU treatment significantly decreased total plasma T3 and T4 concentrations compared with EU controls (Fig. 1, *B* and *C*). The subset of animals treated with PTU for 8 wk and then placed on oral T3 for an additional 2 wk while continuing PTU treatment, had higher T3 levels than the PTU and EU groups, but within the normal reference range. The short half-life of circulating T3 and variation in sampling time from last ingestion likely contributed to the range in T3 values. In the PTU + T3 group of animals, plasma T4 remained suppressed due to the inhibitory effect of PTU on thyroid gland production of T4. This model isolated treatment effects to the exogenously delivered T3. A similar model used in previous studies showed significant changes in heart rate, left ventricular hemodynamics, and echocardiographic measurements (30), and therefore, these parameters were not routinely recorded here.

### Transverse-Tubule Organization

**Figure 2.**
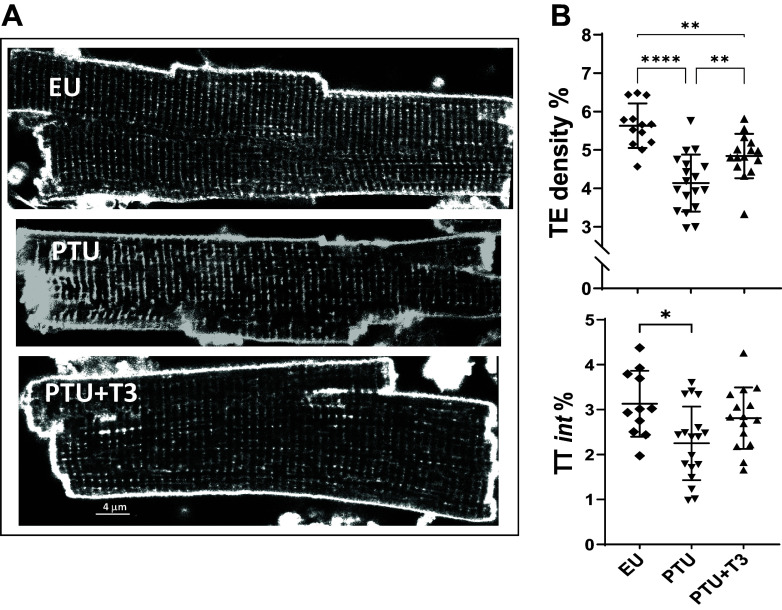
Analysis of T-tubule structures. *A*: representative confocal images of cardiomyocytes from each treatment group (as described in legend of Fig. 1) stained with membrane specific di-8-ANEPPS dye. *B*: AutoTT analysis of transverse-oriented elements (TE) or T-tubules showing percent (%) TE density, and the index of TE-tubule integrity (TT_int_ = TE density × TE regularity). Scatter plots show values of individual cells with group means ± SD of 4–8 cells per heart from 3 animals/group; statistical analysis used one-way ANOVA, post hoc Tukey’s multiple group comparisons; brackets between groups indicate significant differences of *P < 0.05, **P < 0.01, and ****P < 0.0001.

Transverse-tubule elements (TEs) within isolated live cardiomyocytes were stained with di-8-ANEPPS and images were captured by laser-scanning confocal microscopy. Representative images of myocytes derived from animals of each treatment group are shown in Fig. 2*A*. T-tubule analysis using the AutoTT software program showed a significant reduction of TE density and TE integrity (TT*int*) in the PTU-treated hearts, which were increased toward normal values with T3 treatment (Fig. 2*B*). These results are consistent with our prior reports using this disease model (30).

### Ca^2+^ Spark Analysis

**Figure 3.**
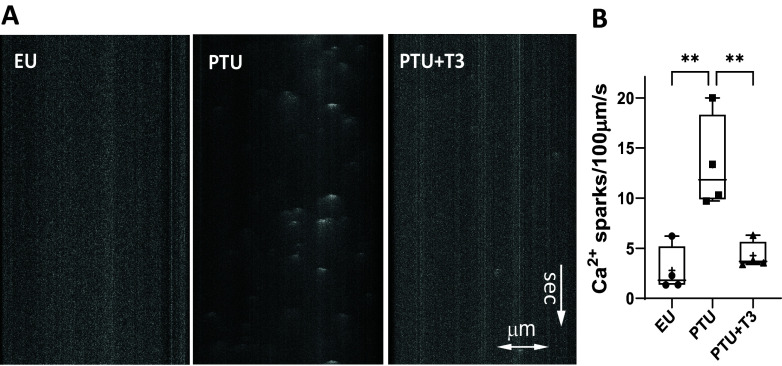
Analysis of calcium sparks in isolated ventricular myocytes. *A*: line-scanned images of representative cardiomyocytes from each study group (panels labeled EU, PTU, and PTU + T3), loaded with the calcium dye Fluo-4 are shown. Spontaneous Ca^2+^ spark images were captured by laser-scanning microscopy in line-scan mode. Scanning line (70 µm; *x*-axis) and scan time (in s; directional arrow in *y*-axis) are identical for all panels. SparkMaster software was used to measure Ca^2+^ spark characteristics. *B*: graph shows number of Ca^2+^ sparks generated over time. Box-and-whisker plot shows median values, box margins at 25th and 75th percentiles, whiskers at maximum/minimum values; each dot is the average value of 10–25 cells/heart; 4 animals in each group. Statistical analysis used one-way ANOVA, post hoc Tukey’s multiple group comparisons. **P < 0.01 between groups as indicated by brackets. EU, euthyroid; PTU, propyl-thiouracil; T3, triiodo-l-thyronine.

Previously, we recorded simultaneous measurements of sarcomeric contractility and Ca^2+^ transients in individual cardiomyocytes and showed that TH deficiency significantly decreased shortening/lengthening velocities that aligned with reduced maximum velocities (±*V*_max_) of the Ca^2+^ transients (30). In the present study, we extended these studies to measure spontaneous Ca^2+^ sparks or leaks in isolated quiescent cardiomyocytes loaded with Fluo4-AM. Representative line-scanned images of cardiomyocytes showing Ca^2+^ sparks are presented in Fig. 3*A*. The total number of Ca^2+^ sparks recorded per cell area over time was significantly higher in PTU cells and was normalized with T3 treatment (Fig. 3*B*). Table 1 lists the Ca^2+^ spark parameters measured using SparkMaster analysis software program. Since the number of Ca^2+^ sparks in individual cardiomyocytes from the same heart varied widely, we combined the spark data from individual cells of all hearts of a treatment group, thus increasing statistical power. Measurements of spark number and spark amplitude (ΔF/F_0_) and the steepness of the spark upstroke [(ΔF/F_0_)/Δ*t*_max_] were significantly increased in TH deficient cells (PTU), and T3 treatment returned these values to normal. The full width (FWHM) and full duration (FDHM) at half-max amplitude were significantly decreased in PTU compared with these spark values in normal EU- and in T3-treated cardiomyocytes.

**Table 1.** Treatment effects on Ca^2+^ spark characteristics in isolated cardiomyocytes

Spark Parameters	EU	PTU	PTU + T3
*n*	5	5	4
Ca sparks/100 µm/s	2.6 ± 0.4	11.4 ± 2.3^a,b^	5.2 ± 0.8
Amplitude (dF/F_0_)	0.876 ± 0.027	2.330 ± 0.069^c^	1.103 ± 0.047
FWHM, µm	1.68 ± 0.06	1.10 ± 0.03^c^	2.34 ± 0.09^d^
FDHM, ms	43.51 ± 2.38	30.07 ± 0.82^c^	53.76 ± 2.60^e^
TtP, ms	29.96 ± 1.87	33.39 ± 1.10	33.04 ± 2.09
(dF/F_0_)/d*t*_max_	63.9 ± 2.0	219.4 ± 8.79^c^	87.9 ± 3.3
Tau	87.35 ± 11.20	78.95 ± 13.58	77.51 ± 5.45

Values are means ± SE; *n*, number of animals/group. SparkMaster analysis generated Ca^2+^ spark measurements, including amplitude [spark fluorescence above fluorescence baseline (dF/F_0_)], full width at half-maximum amplitude (FWHM), full duration at half-maximum amplitude (FDHM), time to peak (TtP), maximum steepness of the spark upstroke [(dF/F_0_)/d*t*_max_], and exponential time constant of the spark decay (tau). Statistical analysis used one-way ANOVA, with post hoc Tukey’s test for multiple group comparisons. EU, euthyroid; PTU, propyl-thiouracil; T3, triiodo-l-thyronine. ^a^*P* < 0.05, PTU vs. PTU + T3; ^b^*P* < 0.0001, PTU vs. EU; ^c^*P* < 0.0001, PTU vs. EU, and PTU + T3; ^d^*P* < 0.0001, PTU + T3 vs. EU; and ^e^*P* < 0.001, PTU + T3 vs. EU.

### Two-Dimensional STORM of RyR2 Clusters and Colocalization of Jph2

**Figure 4.**
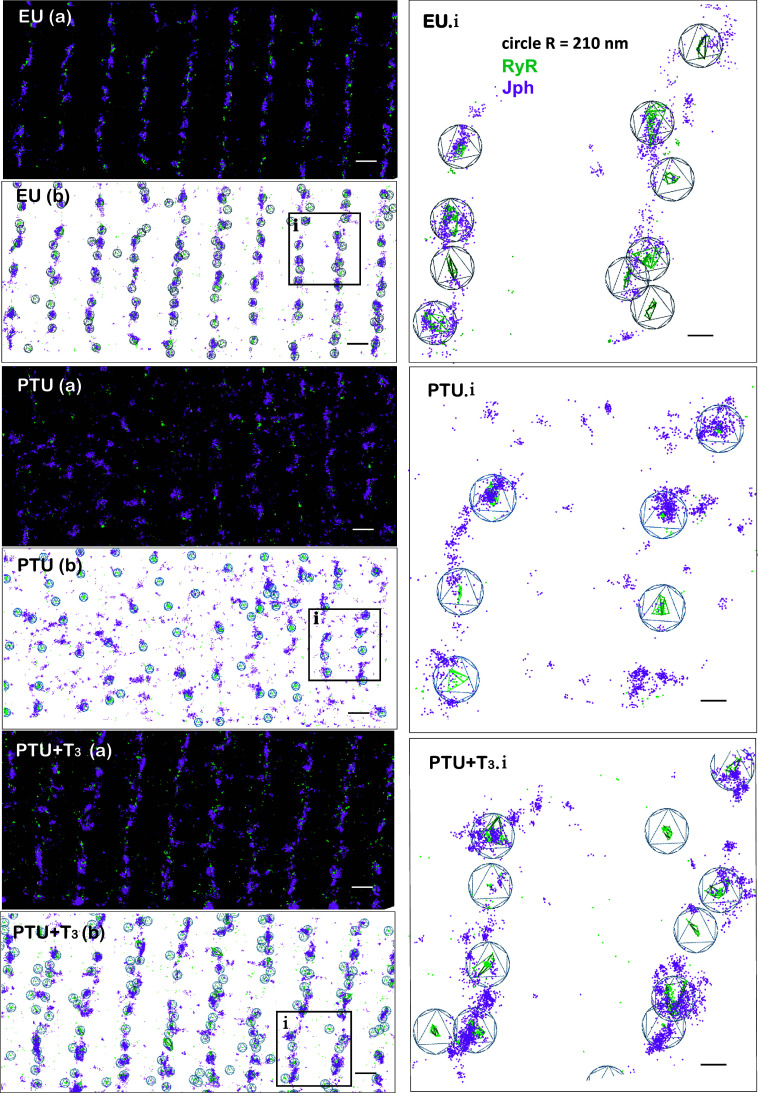
2-D STORM images of cardiomyocytes analyzed for RyR2 clusters and Jph2 localizations. Representative STORM images showing a defined “region of interest” (ROI = 3 × 10^8^ nm^2^) from EU (*a*), PTU (*a*), and PTU + T3 (*a*) cardiomyocytes. Localizations are individual arbitrarily color-coded spots that represent signals in the fluorescent channels corresponding to RyR2 (green) and Jph2 (magenta) immunolabeling. RyR2 and Jph2 localizations show alignment in organized rows corresponding to sarcomere *z*-lines (scale bar, 1 µm). Analysis of localizations in the STORM images of *a* images are shown in the corresponding *b* images EU (*b*), PTU (*b*), and PTU + T3 (*b*) (scale bar, 1 µm). DBSCAN of RyR2 localizations are joined by green mesh, and each cluster is encircled within a 210 nm radius from the cluster centroid (blue circles). Boxed areas (*i*) in each of the *b* images are enlarged in EU.i, PTU.i, and PTU + T3.i (scale bar, 250 nm). RyR2 image data analyses showed that 40%–80% of total RyR2 localizations within a cell area were clustered and that ∼10% to 30% of total Jph2 localized within the defined RyR2 cluster areas. 2-D, two-dimensional; DBSCAN, density-based spatial clustering of applications with noise; EU, euthyroid; Jph2, junctophilin-2; NNDs, nearest neighbor distances; PTU, propyl-thiouracil; ROI, region of interest; RyR, ryanodine receptor; STORM, stochastic optical reconstruction microscopy; T3, triiodo-l-thyronine.

Representative two-dimensional (2-D) STORM images of cardiomyocytes from each treatment group are shown in Fig. 4, images EU (*a*), PTU (*a*), and PTU + T3 (*a*), showing individual color-coded localizations (or spots) corresponding to RyR2 (green) and Jph2 (magenta) proteins. Identification of clustering of RyR2 localizations was determined by a hierarchical density-based algorithm using a search distance of 100 nm and requiring a minimum of 10 RyR2 localizations to define a cluster and is indicated by green-colored lines joining RyR2 localizations. We observed that cluster size, defined as the distance between the furthest two RyR2 localizations within clusters, averaged 235 ± 38 nm. A circular area outlined in blue color with a radius of 210 nm from the cluster centroid was used to quantify the Jph2 localizations associated with each cluster. The resulting cluster and localization analysis of the STORM images in the *a* images are illustrated in the corresponding images in Fig. 4 EU (*b*), PTU (*b*), and PTU + T3 (*b*). Clearly evident is the highly organized alignment of rows of RyR2 clusters and colocalized Jph2 in the EU and PTU + T3 myocytes, while significant disarray of these proteins can be observed in the thyroid-deficient PTU-treatment condition. These observations correspond to the degree of organization of *t* tubules visualized by ANEPPS staining in Fig. 2*A*, and we propose that the encircled RyR2 clusters align with TT-jSR dyad structures. Higher magnification of the boxed areas (*i*) in each of the *b* images is shown in images EU.i, PTU.i, and PTU + T3.i.

**Figure 5.**
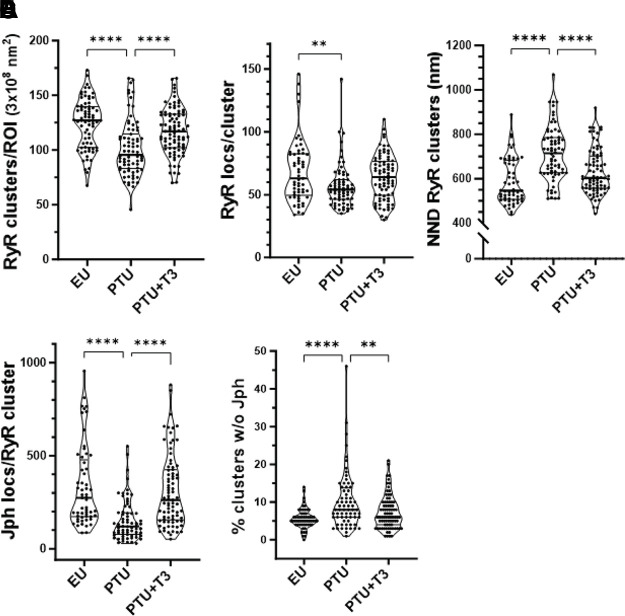
Analysis of 2-D STORM images of RyR2 clusters and Jph2 colocalizations. Comparison of cluster numbers and localizations among treatment groups (EU, PTU, and PTU + T3) shown as violin plots indicating group median and quartiles (thick and thin horizonal lines) (individual points are averaged values of 2 ROIs per cell from 10 to 12 cells per heart from 5 or 6 animals per group). *A*: total number of RyR2 clusters per ROI identified by DBSCAN. *B*: number of RyR2 localizations in each cluster. *C*: NNDs between RyR2 clusters. *D*: number of Jph2 localizations quantified within the 210 nm search radius of each RyR2 cluster centroid. *E*: percentage of RyR2 clusters with no Jph2 localizations within the 210 nm search radius. Statistical analysis by one-way ANOVA, post hoc Tukey’s multiple comparisons test; *P* values between groups indicated by brackets. 2-D, two-dimensional; DBSCAN, density-based spatial clustering of applications with noise; EU, euthyroid; Jph2, junctophilin-2; NNDs, nearest neighbor distances; PTU, propyl-thiouracil; ROI, region of interest; RyR, ryanodine receptor; STORM, stochastic optical reconstruction microscopy; T3, triiodo-l-thyronine. **P < 0.01, ***P < 0.001, and ****P < 0.0001.

Quantitation of total RyR2 clusters within a defined cell area (ROI of 3.0 × 10^8^ nm^2^) showed that thyroid deficiency (PTU) resulted in a significant decrease in cluster number compared with EU control and that this was subsequently normalized by T3 treatment alone (PTU + T3) (Fig. 5*A*). Furthermore, the number of RyR2 localizations within each cluster was significantly reduced in the PTU cardiomyocytes with a return toward euthyroid values with T3 treatment (Fig. 5*B*). As might be expected with the disorganization of T-tubules with thyroid deficiency, the nearest neighbor distances (NNDs) measured between RyR2 cluster centroids were significantly greater in the PTU condition compared with either control EU or T3-treated myocytes (714 ± 15 vs. 588 ± 14 or 630 ± 11 nm, respectively) (Fig. 5*C*). Most notably, the number of Jph2 localizations closely associated with RyR2 clusters (within the 210 nm radius) was significantly reduced in the PTU-treated cardiomyocytes, while T3 treatment of the thyroid-deficient animals completely reversed this effect (Fig. 5*D*). Although Jph2 localizations were concentrated at RyR2 clusters, these proteins were also observed outside the cluster search areas (Fig. 4). The STORM images support the observation that the majority of Jph2 align with T-tubules along z-lines in normal cells, and that this arrangement is disrupted in thyroid deficiency. We noted that the number of RyR2 clusters without associated Jph2 was significantly higher in PTU cells compared with controls (10 ± 1 vs. 5.6 ± 0.4%), and this percentage was lowered to 7 ± 0.5% (*P* < 0.01) with T3 treatment (Fig. 5*E*).

**Figure 6.**
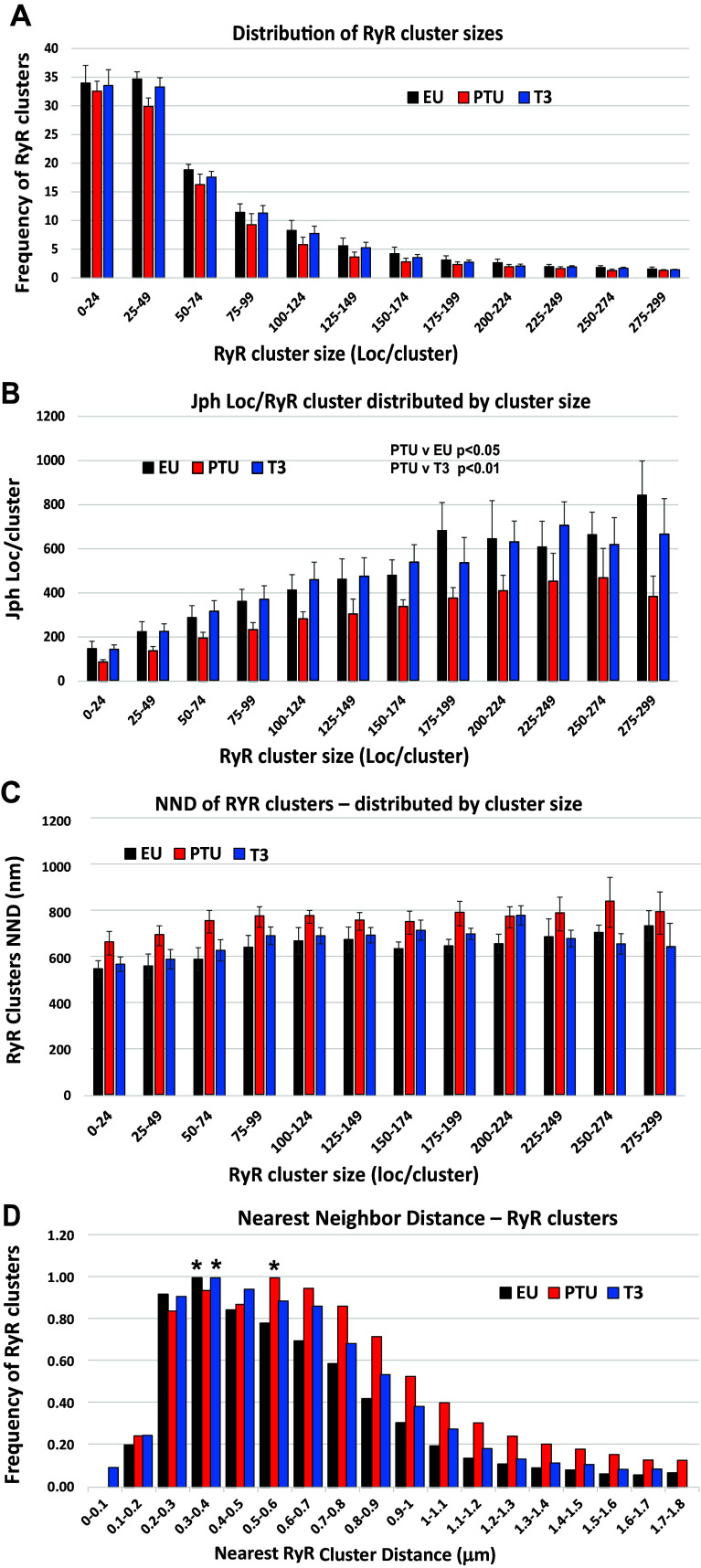
Histograms showing the distribution of RyR2 clusters by size. STORM images were analyzed by DBSCAN to identify RyR2 clusters and number of Jph2 colocalized with RyR2 clusters. Histogram shows RyR2 clusters segregated by size into bins of 25 localizations per cluster from 10 minimum localizations (loc) to 299 loc/cluster (displayed on *x*-axis). Bar graphs represent means ± SE of data averaged from 10 to 12 cells/heart of 5 to 6 animals per group [EU, PTU, and PTU + T3 (T3)]. Statistical analysis of data in each histogram used a linear mixed-effects model with Tukey’s post hoc pairwise comparisons. *A*: bars represent the number of RyR2 clusters (*y*-axis) in each bin. *B*: Jph2 localizations per cluster binned by RyR2 cluster size; LME statistical analysis showed differences between PTU vs. EU (*P* < 0.05) and PTU vs. T3 (*P* < 0.01). *C*: NNDs between RyR2 clusters based on cluster size. *D*: relative frequencies of RyR2 clusters (*y*-axis) distributed by NND between clusters (*x*-axis). *NND with the highest number of RyR2 clusters from each group with majority of RyR2 clusters from EU and T3 myocytes measuring 0.3–0.4-µm NND, whereas NND of clusters in PTU cells was 0.5–0.6 µm. DBSCAN, density-based spatial clustering of applications with noise; EU, euthyroid; Jph2, junctophilin-2; LME, linear mixed-effect; NNDs, nearest neighbor distances; PTU, propyl-thiouracil; ROI, region of interest; RyR, ryanodine receptor; STORM, stochastic optical reconstruction microscopy; T3, triiodo-l-thyronine.

Although RyR2 cluster sizes ranged from 10 to greater than 300 localizations (locs), the occurrence or frequency of these various sizes varied. To determine this size distribution, we binned the clusters into 25 locs per bin, as plotted in histograms in Fig. 6. Approximately 70% of all clusters contained between 10 (preset minimum defined as a cluster) to 49 localizations, with ∼15% falling into the 50–74 locs, and the remainder falling into progressively larger cluster sizes in smaller numbers (Fig. 6*A*). Notably, regardless of cluster size at least up to ∼150 localizations per cluster, the frequency of clusters in the PTU myocytes tended to be less than in either EU or T3-treated conditions, and this correlates with the median RyR2 locs/cluster shown in the violin plots of Fig. 5*B*.

We similarly determined the distribution of Jph2 per cluster based on the binned RyR2 cluster sizes (Fig. 6*B*). Two observations were noted: *1*) the number of Jph2 localizations increased as the RyR2 cluster size increased and *2*) there were significantly fewer Jph2 locs in every cluster size in the PTU group of animals. Statistical analysis using a linear mixed-effects (LMEs) model showed significant differences among study groups in the Jph locs/RyR2 cluster histogram datasets (Fig. 6*B*) with an adjusted *P* value of 0.036 between EU and PTU groups, and *P* = 0.008 between PTU and PTU + T3 groups.

NND between RyR2 clusters based on their size is shown in the histogram of Fig. 6*C*. We did not find any statistical differences among the study groups using the LME model; however, there was a clear trend of increased distances between RyR2 clusters in the PTU cells regardless of the size of the cluster. These data corroborate the significant results observed when comparing the median NND values of the three groups, as shown in Fig. 5*C*. Expressing the distribution of RyR2 clusters based on their NND (Fig. 6*D*), we found that in the EU- and T3-treated cells, the largest number of clusters fell within 300–400 nm of each other, whereas in the PTU cells, the majority of clusters were 500–600 nm apart. This cluster distribution clearly indicates a rightward shift to greater neighboring cluster distances in the PTU cardiomyocytes.

### STORM Imaging and Analysis of RyR2 Clusters, Ca_V_1.2, and Jph2

**Figure 7.**
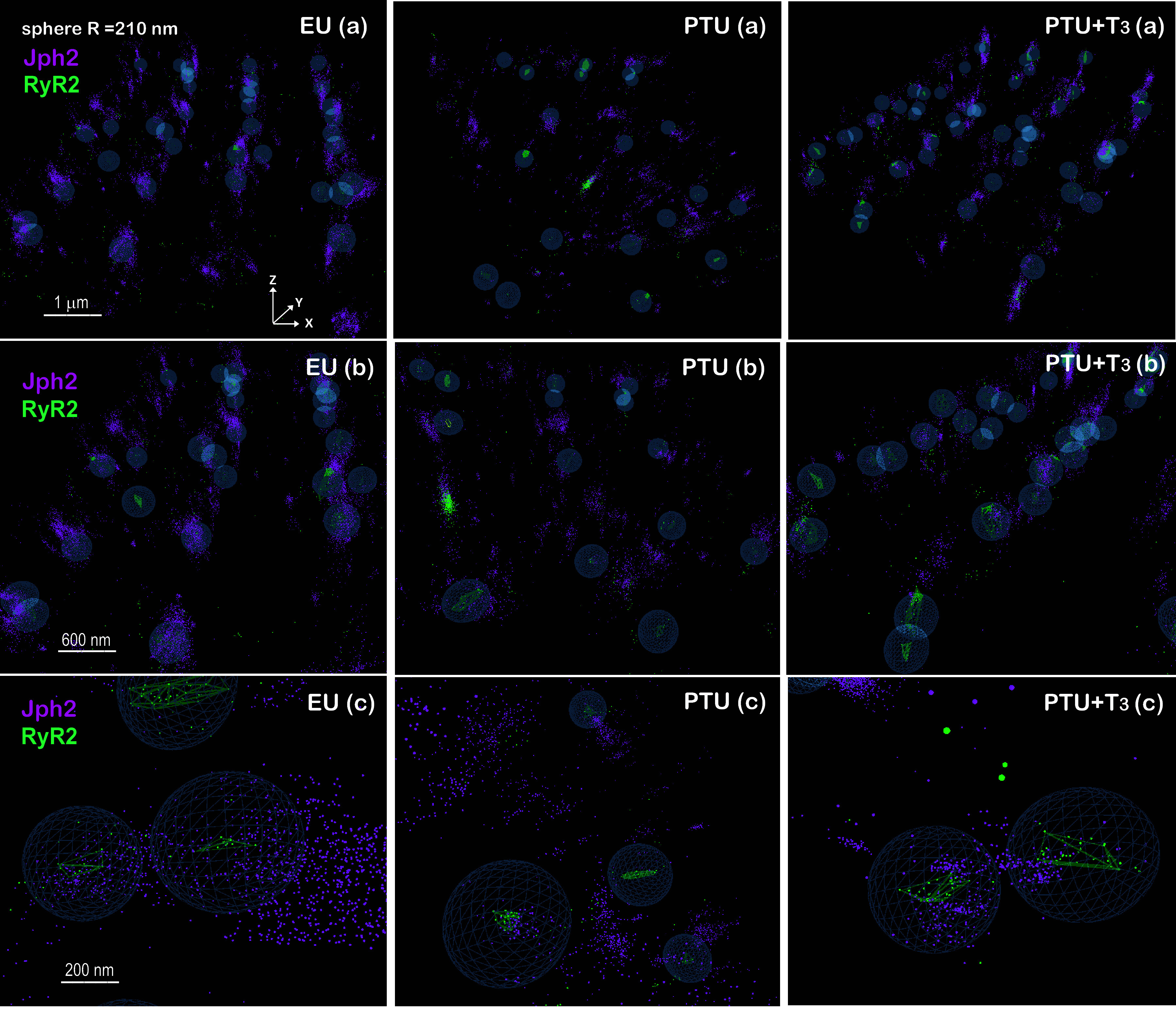
3-D STORM images of RyR2 clusters and Jph2 localizations in ventricular myocytes. Representative 3-D STORM images of cardiomyocytes isolated from EU, PTU, and PTU + T3-treated animals. Localizations are individual arbitrarily color-coded spots that represent signals in the fluorescent channels corresponding to RyR2 (green) or Jph2 (magenta). *a*: subregions of an analyzed ROI (3 × 10^11^ nm^3^) are shown to illustrate a 3-D view of the image [*x*, *y*, *z* planes indicated in EU (*a*)] with RyR2 clusters encircled by a sphere (blue lattice) measuring 210 nm radius from the cluster centroid, and individual Jph2 are seen as spots or localizations (scale bar is 1 µm at the front of the 3-D image). *b*: images are higher magnification of regions in corresponding images in *a* images (scale bar, 600 nm) illustrating RyR2 clusters (spots joined by green mesh) and Jph2 (magenta spots) inside and outside each sphere encircling a cluster. *c*: images illustrate higher magnification of individual RyR2 clusters within each sphere colocalized with Jph2 (scale bar, 200 nm). 3-D, three-dimensional; EU, euthyroid; Jph2, junctophilin-2; PTU, propyl-thiouracil; ROI, region of interest; RyR, ryanodine receptor; STORM, stochastic optical reconstruction microscopy; T3, triiodo-l-thyronine.

**Figure 8.**
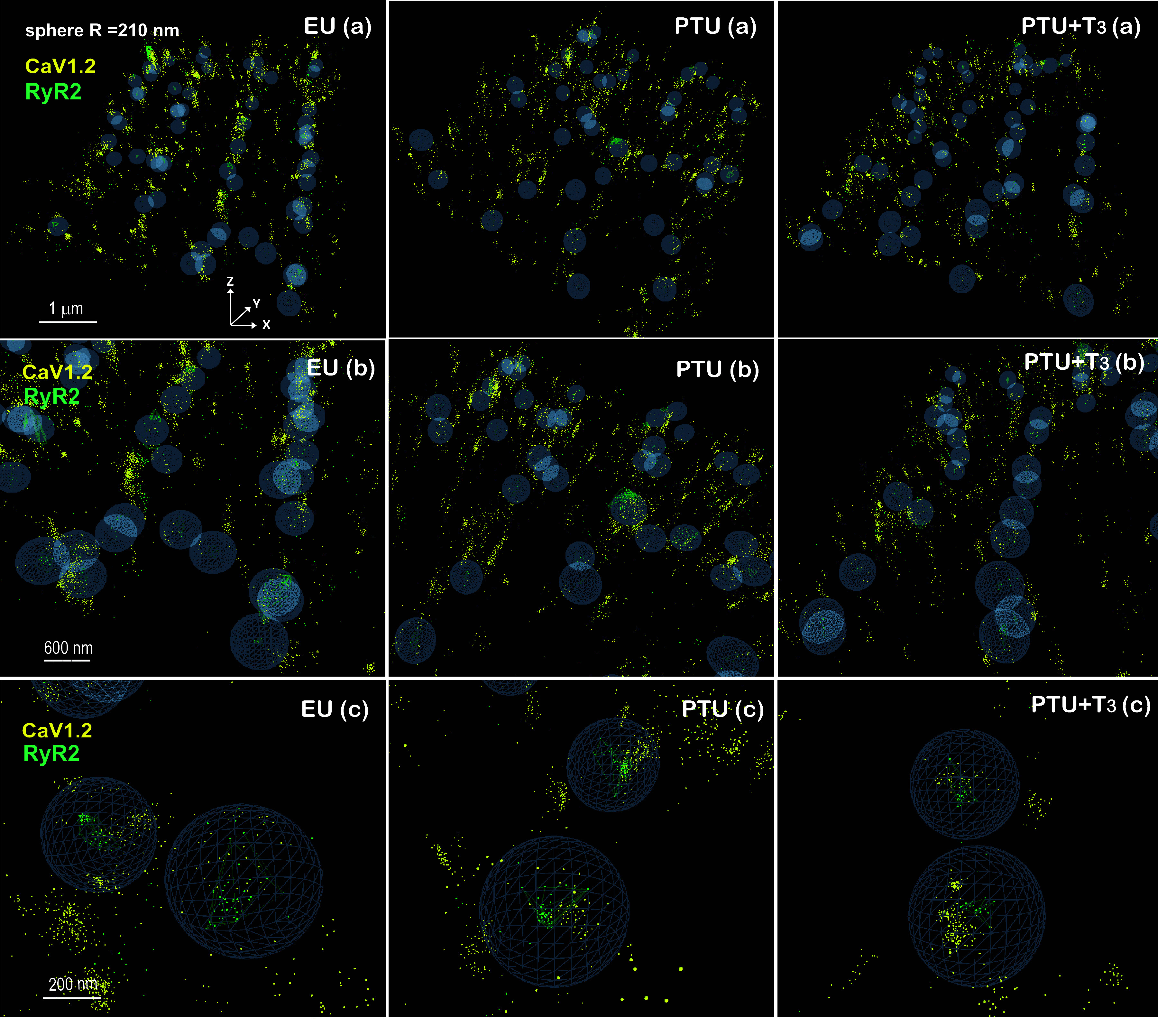
3-D STORM images of RyR2 clusters and Ca_V_1.2 in ventricular myocytes. Representative 3-D images of cardiomyocytes from the three study groups (EU, PTU, and PTU + T3) are illustrated as described in legend to Fig. 7 showing localizations of RyR2 (green mesh) clustered within 210 nm radius spheres (blue lattice). Localizations of Ca_V_1.2 (yellow spots) appear within and outside the spheres. Images in *b* and *c* are magnified subregions of the ROIs shown in corresponding *a* images. Scale bars are indicated. 3-D, three-dimensional; EU, euthyroid; PTU, propyl-thiouracil; ROI, region of interest; RyR, ryanodine receptor; STORM, stochastic optical reconstruction microscopy; T3, triiodo-l-thyronine.

3-D STORM imaging provided additional insight into the spatial organization of L-type calcium channels (Ca_V_1.2) and Jph2 associated with RyR2 clusters. One limitation was the short axial range (∼1.5 µm) captured in these 3-D STORM images that were obtained within the cell interior. Similar to 2-D image analysis, the 3-D localization data were subject to the same DBSCAN parameters to define RyR2 clusters and the number of localizations of Ca_V_1.2 and Jph2 within a sphere of 210 nm radius from the RyR2 cluster centroid. Analyzed 3-D images of cardiomyocytes from each study group are presented in Fig. 7, with *x*, *y*, and *z* axes as labeled in EU (*a*). By tilting each 3-D image such that the *z*-axis is observed, the three-dimensional aspect of the image (*x*, *y*, *z* planes) can be appreciated. In Fig. 7, EU (*a*), PTU (*a*), and PTU + T3 (*a*) show a subregion of an analyzed ROI (3.0 × 10^11^ nm^3^) with RyR2 clusters (green mesh) encircled by a sphere (blue cage), and with Jph2 localizations (magenta spots). In Fig. 7, *b* and *c* images represent higher magnifications of regions in the corresponding a images (scale bars apply to the front of the image). As with the 2-D images, the highly organized alignment of the RyR2 and Jph2 proteins is readily apparent in the EU and T3-treated cells, whereas these proteins exhibit significant disorganization in the PTU condition. RyR2 and Jph2 proteins in normal cells appear to colocalize, whereas RyR2 clusters in PTU myocytes are fewer in number and have fewer associated Jph2, which appear to localize largely outside the search area or sphere. Similar to the analysis of Jph2 association with RyR2 clusters, we analyzed how Ca_V_1.2 localizes with RyR2 clusters. Representative 3-D images of cardiomyocytes after LumeVR DBSCAN analysis for Ca_V_1.2 localizations (yellow spots) and RyR2 clusters (green mesh within a blue sphere) are shown in Fig. 8 (labeling and magnification are as described in Fig. 7).

**Figure 9.**
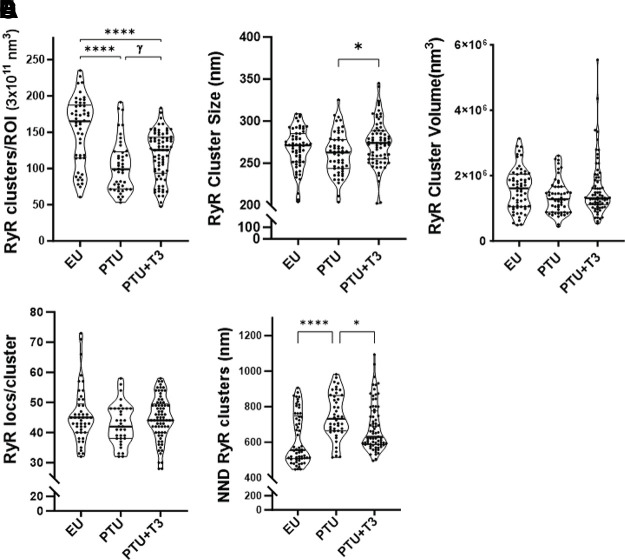
Analysis of RyR2 clusters in captured 3-D STORM images. Comparison of RyR2 clusters among study groups (EU, PTU, and PTU + T3) presented as violin plots showing group median (thick line) with first and third quartiles (thin horizonal lines) (individual points are averaged values of 2 ROIs per cell from 8 to 10 cells per heart from 5 animals per group). *A–C*: total number of RyR2 clusters per ROI (volume) (*A*), RyR2 cluster size is the distance between the furthest two RyR2 localizations in the cluster (*B*), and RyR2 cluster volume created by connecting the perimeter RyR2 localizations in the cluster (*C*; 3-D mesh in Fig. 8). *D*: number of RyR2 localizations per cluster analyzed by DBSCAN. *E*: NNDs between RyR2 clusters. Statistical analysis used one-way ANOVA, post hoc Tukey’s multiple comparisons test; *P* values between groups indicated by brackets. *P < 0.05 and ****P < 0.0001. Statistical analysis between PTU and PTU + T3 used unpaired *t* test with Welch’s correction, γ*P* < 0.001. 3-D, three-dimensional; DBSCAN, density-based spatial clustering of applications with noise; EU, euthyroid; NNDs, nearest neighbor distances; PTU, propyl-thiouracil; ROI, region of interest; RyR, ryanodine receptor; STORM, stochastic optical reconstruction microscopy; T3, triiodo-l-thyronine.

Quantitation of image data represents the average values obtained from two equal ROI volumes (3.0 × 10^11^ nm^3^) per cell. The total number or density of RyR2 clusters in PTU cardiomyocytes was significantly reduced compared with EU controls, and while T3 treatment increased cluster numbers, these numbers remained lower than control values but were significantly higher than PTU when statistical comparison was made only between the two groups (Fig. 9*A*). The median cluster number in the 3-D image analysis approximates those quantified in the 2-D images (Fig. 5*A*), although the quartile values are greater. The RyR2 cluster size measured as the distance (nm) between the furthest two localizations was marginally decreased in the PTU cells while significantly increased with T3 treatment (Fig. 9*B*). The volumes of RyR2 clusters were similar among the study groups (Fig. 9*C*). In contrast to 2-D image analysis, the number of RyR2 localizations per cluster remained largely unchanged by treatment (Fig. 9*D* compared with Fig. 5*B*). Notably, nearest neighbor distances between RyR2 clusters (NND) were significantly greater in the PTU myocytes [median (25–75%tile)] of 732 nm (663–863) versus EU of 556 nm (508–748), with T3 treatment having a significant effect by decreasing distances between clusters to 630 nm (587–776) (Fig. 9*E*).

**Figure 10.**
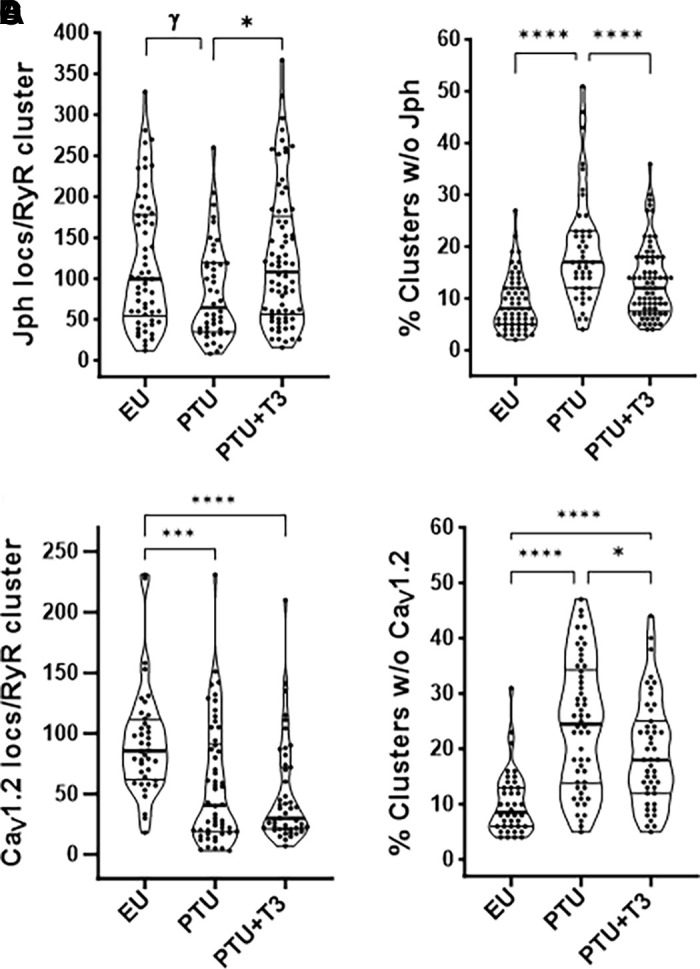
Jph2 and Ca_V_1.2 localizations per RyR2 cluster analyzed in 3-D STORM images. Quantitation of Jph2 and Ca_V_1.2 localizations within a sphere of 210 nm radius of the RyR2 cluster centroid are shown as violin plots as described in legend to Fig. 9. *A*: Jph2 localizations within the RyR2 cluster sphere. *B*: percentage of total RyR2 clusters in the cell area measured (ROI volume) without any associated Jph2. *C*: Ca_V_1.2 localizations per cluster sphere. *D*: percentage of all clusters without Ca_V_1.2. Statistical analysis comparing study groups (EU, PTU, and PTU + T3) used one-way ANOVA, post hoc Tukey’s multiple comparisons test; *P* values between groups indicated by brackets. γ*P* = 0.06, *P < 0.05, ***P < 0.001, and ****P < 0.0001. 3-D, three-dimensional; EU, euthyroid; Jph2, junctophilin-2; PTU, propyl-thiouracil; ROI, region of interest; RyR, ryanodine receptor; STORM, stochastic optical reconstruction microscopy; T3, triiodo-l-thyronine

Quantitation of Jph2 and Ca_V_1.2 localizations within a 210 nm radius sphere of the RyR2 cluster centroids are shown in Fig. 10. Similar to 2-D image analysis, the 3-D images showed significant decreases in Jph2 localizations per cluster in PTU myocytes, and normalization with T3 treatment (Fig. 10*A*). As might be expected from published studies comparing 2-D and 3-D STORM image analysis (43), the current study showed median Jph2 localization values that were two- to threefold lower in 3-D compared with 2-D images (Fig. 5*D*). The number of Ca_V_1.2 localizations per cluster was also significantly reduced in the thyroid deficient PTU cells, but this number remained decreased in the T3-treated cardiomyocytes (Fig. 10*C*). The number of RyR2 clusters without any associated Jph2 or Ca_V_1.2 increased twofold (*P* < 0.001) in the PTU myocytes, with significant reductions toward control values in the T3-treated hearts (Fig. 10, *B* and *D*). Since images of RyR2 clusters were taken within the cell interior, we may assume that some clusters without Ca_V_1.2 had reorganized into nondyadic pools that reflect the disarray of the T-tubule networks in the thyroid deficient cardiomyocytes.

**Figure 11.**
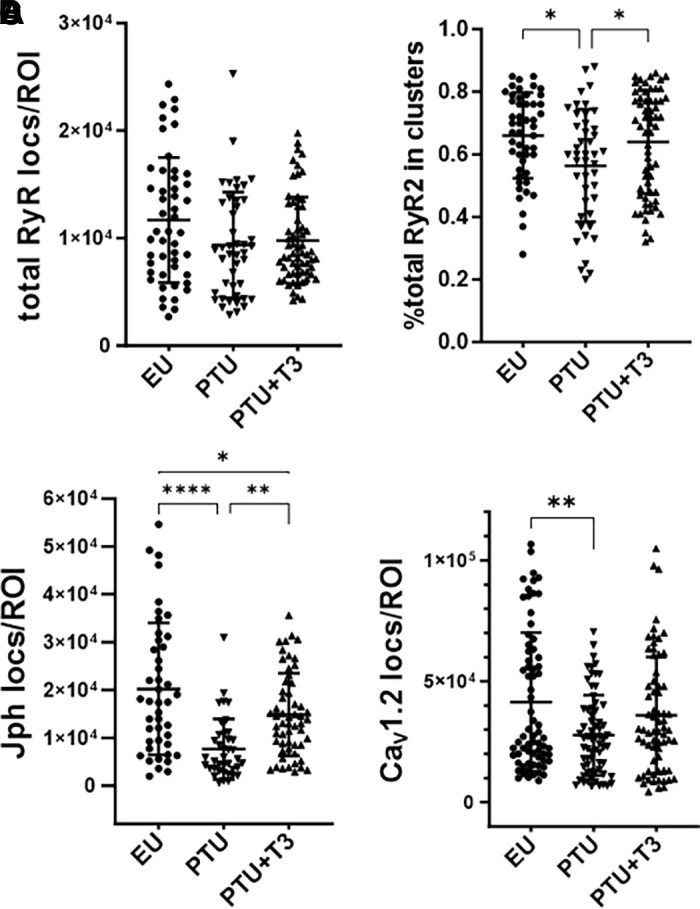
3-D STORM image analysis of cardiomyocyte RyR2, Jph2, and Ca_V_1.2 channels. Cell area or ROI in 3-D longitudinal images of cardiomyocytes covered 10 sarcomeres and measured 3 × 10^11^ nm^3^. Scatter plots show individual values averaged from 2 ROIs of 8–10 cells/heart from 5 animals/group. Means ± SD are shown for each group (EU, PTU, and PTU + T3). *A*: total RyR2 localizations in each ROI. *B*: percentage of total RyR2 in each ROI that were clustered by DBSCAN analysis. *C* and *D*: total number of Jph2 (*C*) and Ca_V_1.2 (*D*) localizations within a 210-nm sphere of the RyR2 cluster centroid. Statistical analysis of group means used one-way ANOVA, post hoc Tukey’s multiple group comparisons. 3-D, three-dimensional; DBSCAN, density-based spatial clustering of applications with noise; EU, euthyroid; Jph2, junctophilin-2; PTU, propyl-thiouracil; ROI, region of interest; RyR, ryanodine receptor; STORM, stochastic optical reconstruction microscopy; T3, triiodo-l-thyronine. *P < 0.05, **P < 0.01, and ****P < 0.0001.

To further characterize the organization of these ion channels, we measured total localization counts in a cell area or ROI, referred to as density. Total RyR2 localizations or density tended to be ∼17% to 20% lower in PTU myocytes compared with EU- and T3-treated cells (Fig. 11*A*), and significantly fewer RyR2 localizations were found in clusters in the PTU cells, suggestive of RyR2 tetramers residing outside dyadic structures (Fig. 11*B*). Furthermore, we observed that total Jph2 and total Ca_V_1.2 localization numbers in the ROI were significantly lower in the PTU myocytes, and that T3-treatment normalized Jph2 but did not alter Ca_V_1.2 (Fig. 11, *C* and *D*).

**Figure 12.**
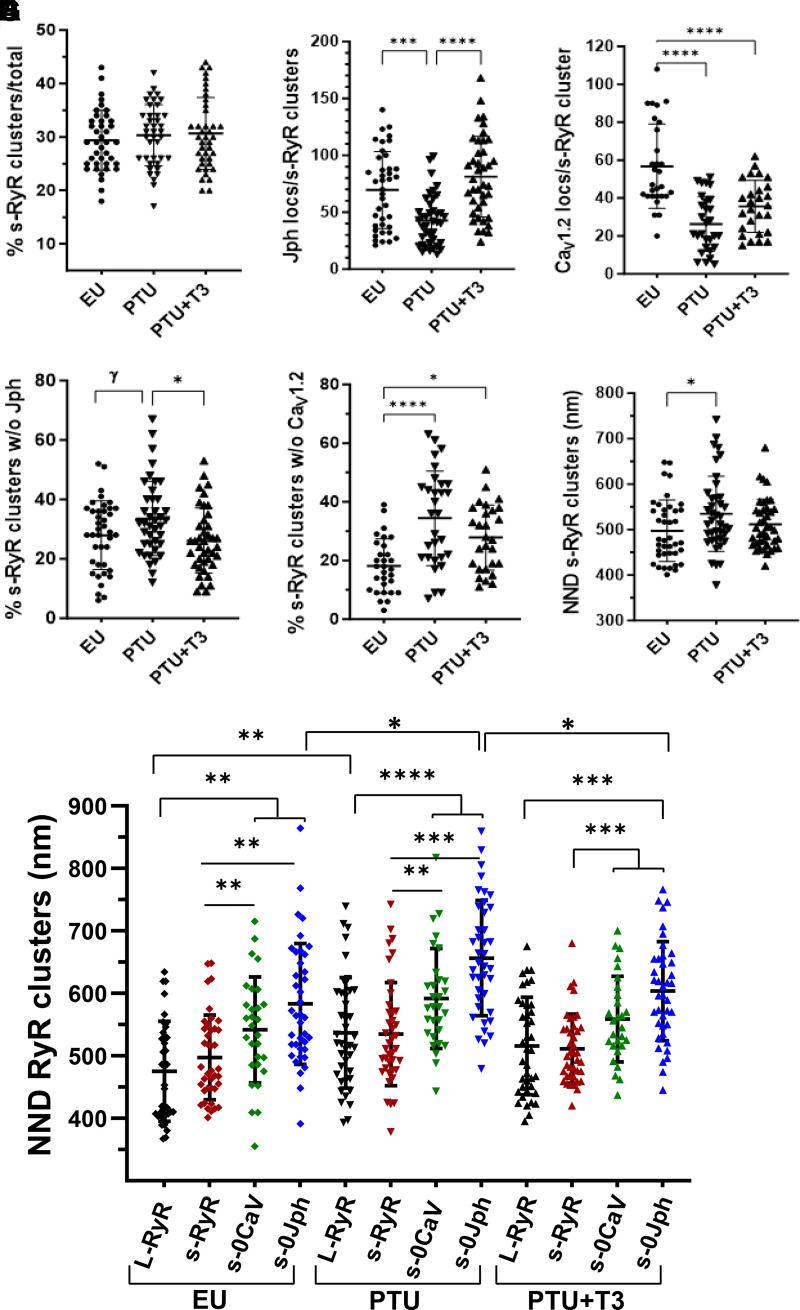
3-D STORM image analysis of small RyR2 clusters comprising of 4–9 localizations. Quantitation of RyR clusters, Jph2, and Ca_V_1.2 localizations are as described in Figs. 9 and 10 legends. Scatter plots are individual values of 8 cells/heart from 3 animals/group. Means ± SD are shown for each group (EU, PTU, and PTU + T3). *A*: small RyR (s-RyR) clusters as percentage of total RyR clusters per ROI. *B* and *C*: total number of Jph (*B*) and Ca_V_1.2 (*C*) localizations per s-RyR cluster. *D* and *E*: percentage of s-RyR clusters without associated Jph2 (*D*) or Ca_V_1.2 (*E*). *F*: NNDs between s-RyR clusters in each study group. *G*: comparisons of NNDs among RyR clusters; L-RyR are large clusters of ≥10 localizations/cluster; s-RyR are small clusters (4–9 locs); s-0Cav are s-RyR without Ca_V_1.2; s-0Jph are s-RyR without Jph2. Statistical analysis used one-way ANOVA, post hoc Tukey’s multiple group comparisons; lines/brackets between groups indicate significance at *P < 0.05, **P < 0.01, ***P < 0.001, and ****P < 0.0001. 3-D, three-dimensional; EU, euthyroid; Jph2, junctophilin-2; NNDs, nearest neighbor distances; PTU, propyl-thiouracil; ROI, region of interest; RyR, ryanodine receptor; STORM, stochastic optical reconstruction microscopy; T3, triiodo-l-thyronine.

We examined whether RyR2 clusters smaller than 10 localizations might be nondyadic as has been described in human idiopathic dilated cardiomyopathy myocytes (26). Small RyR2 clusters (s-RyR) in the 3-D STORM images were identified as containing four to nine localizations, and these represented ∼30% of the total RyR2 clusters in the three study groups (Fig. 12*A*). Similar to larger RyR2 clusters with ≥10 localizations (labeled here as L-RyR), these s-RyR clusters showed similar results of treatment with PTU myocytes having significantly fewer colocalized Jph2 and Ca_V_1.2 than EU cells and exhibited increases with T3 treatment (Fig. 12, *B* and *C*). Notably, however, ∼30% of these s-RyR clusters had no associated Jph2 compared with only 10% of L-RyR clusters, and similarly, ∼20% of s-RyR were not associated with Ca_V_1.2 (Fig. 12, *D* and *E*). Whether any of these s-RyR clusters lacked both Jph2 and Ca_V_1.2 would require three-color imaging. These smaller clusters were further away from their nearest neighbor (Fig. 12*F*), and this was specifically evident in clusters without associated Jph2 or Ca_V_1.2 (Fig. 12*G*). This NND pattern was similar for all treatment groups, inferring that many small RyR clusters without Jph or Ca_V_1.2 may be extradyadic with greater numbers of these clusters in thyroid-deficient PTU myocytes. These observations of altered cluster size and density reflect those reported in IDCM myocytes in which Hou and colleagues (26) suggest that loss of RyR tetramers and reduced cluster size at dyads in IDCM could lessen the potential of RyR to activate, and the increased distances between RyR clusters would reduce the ability to generate an efficient Ca^2+^ transient.

## DISCUSSION

Our primary conclusions from ultrastructural and single-molecule imaging are that thyroid hormones, specifically bioactive T3, have a regulatory role in maintaining the organization of RyR2 and LTCC within dyadic structures; and, second, that T3 regulates the colocalization of Jph2 within RyR2 clusters. In our prior studies, RNA sequencing and RT-PCR studies in vivo and in cultured adult cardiomyocytes showed that T3 increased expression of Jph2 (20, 30). Canonical thyroid response elements have not been identified in the Jph2 gene, but several studies have reported Jph2 regulation by microRNAs (44, 45). Whether T3 uses this regulatory mechanism in Jph2 expression is currently not known. Jph2 functions primarily as a structural protein spanning T-tubule membranes and jSR at dyad structures involved in EC coupling, and it has been proposed as a druggable target in ameliorating deficiencies of cardiac function in aging and heart disease (28, 46, 47). In light of our numerous prior investigations of the potential therapeutic role of THs in heart failure, we focused this study on the effects of T3 on Jph2 localization with RyR2 clusters and organization within the dyad using a TH-deficient disease model. The importance of normal thyroid status in heart health cannot be overstated since thyroid hormones directly regulate over 500 protein-coding genes expressed in the myocardium, and indirectly control other cellular processes through noncoding RNAs (miRNAs, lncRNAs, circRNAs, etc.) and by nongenomic signaling mechanisms involved in protein synthesis, mitochondrial respiration, and vasoreactivity (reviewed in Refs. 1, 2, 4, 5, and 48). As early as 1986, Dulhunty et al. (49) reported effects of thyroid hormone on the volume and surface area of T-tubules and terminal cisternae that altered membrane capacity. More recently developed nanoscale imaging technologies have illuminated the organization of ryanodine receptor clusters at dyad structures, and single-molecule microscopy has shown that the distribution of ion channels is highly dynamic and regulated (41, 42). The ultrastructural remodeling in TT-jSR membranes and ion channel organization that occurs in heart failure involves complex processes, and many mechanisms are likely responsible. Imaging studies of failing human hearts have shown T-tubule remodeling into sheet-like structures with impaired calcium dynamics, and altered organization of RyR2 clusters and Jph2 (21, 26, 47).

### STORM Imaging of RyR2 Clusters

Optical superresolution imaging has been instrumental in characterizing RyR2 organization in clusters and informing how changes in clustering can modify calcium release and, thereby, cardiac contractile activity (41, 43). Jayasinghe and colleagues (41) used DNA-PAINT imaging with <10 nm precision to visualize the clustering of single RyR2 tetramers. Results showed RyR2 in irregular clustering patterns colocalized with Jph2 molecules with an estimated average RyR2 cluster size of 8.8 ± 3.56 (means ± SE) tetramers. More recently, these authors used 3-D MINFLUX imaging to characterize RyR2 organization at ∼3 nanometer resolution, and by focusing on surface sarcolemma couplings, they estimated 18.6 ± 4.1 RyR2s for clusters with ≥2 RyR2 subunits, and 7.91 ± 1.17 RyR2s for all clusters (50). In the present study using 3-D STORM imaging, we estimated ∼20 to 30 nm resolution of a fluorophore location. Since bound antibody can be at any angle, the actual location of a RyR2 tetramer within a cluster would not be resolved. Although individual puncta within a RyR2 cluster may not be resolvable, a cluster of a minimum of three RyR2 tetramers comprising 12 epitopes would theoretically bind between 3 to 12 monoclonal antibodies linked to a fluorophore. We carried out DBSCAN clustering with the minimum localization number set to 10 to define a RyR2 cluster, and as discussed later herein, we compared these results to clusters comprising RyR2 localizations between 4 and 9.

Grouping RyR2s into clusters has relied on using a threshold distance of 30–50 nm for clusters and 100–150 nm for superclusters (40). In the present study, using a DBSCAN radius of 100 nm to cluster RyR2 localizations, we measured 69 ± 25 and 44 ± 8 (means ± SE) localizations per cluster in 2-D and 3-D images of normal cells, respectively. In our earlier work studying effects of thyroid deficiency on TTs and ion channel organization, similar 2-D STORM images were analyzed using hierarchical density-based clustering of RyR2 localizations with further constraints applied to cluster size (30). Present study advances include the use of conjugated RyR2-DyLight 550 primary antibodies compared with labeled secondary antibodies and the utility of the LumeVR data analysis platform. Nevertheless, results from these studies confirm that hypothyroid PTU-treated cardiomyocytes had significantly reduced density or number of RyR2 clusters per cell area and that RyR2 localizations per cluster were decreased compared with control EU myocytes, which we contend reflects the disarray of the TT ultrastructure and reduction of functional dyads. T3 treatment normalized cluster numbers per cell and increased RyR2 localizations per cluster; however, as we discuss later herein, results from 3-D imaging showed only marginal effects of treatment on RyR2 cluster size.

RyR2 cluster geometry depends not only on the number of RyR2 tetramers but also on their packing density and arrangement to each other within the cluster (51). Cluster size can be inferred by dSTORM imaging that generates localization data from the rate and detection of single-molecule photo-switching event counts. Although we were unable to resolve the internal arrangement of RyR2 tetramers within a cluster, the 30 nm resolution allowed localization of the boundary of the cluster such that the determination of the centroid of each cluster approximated that determined by DNA-PAINT. Since specific experimental protocols critically impact the generation of single-molecule events, only relative changes among experimental groups are valid comparisons. We argue that the imaging results in the present study be considered as relative measurements among study groups.

### Two-Dimensional Imaging of Jph2 Localization with RyR2 Clusters

One of several functions assigned to Jph2 is its role in tethering dyad T-tubules and jSR membranes, thereby juxtaposing RyR2 and Ca_V_1.2 channels for effective EC coupling (52, 53). Guo et al. (54) have recently shown that calpain-mediated cleavage of Jph2 under cardiac stress conditions resulted in loss of dyad structures and EC uncoupling. Furthermore, cleavage of Jph2 at its COOH-end released an NH_2_-terminal fragment that translocated to the nucleus and associated with chromatin to regulate transcription. The COOH-terminus of Jph2 contains the SR membrane-anchoring transmembrane (TM) domain shown to interact directly with RyR2. The Jph2 antibody used in the present study targets this COOH-terminal region, and we verified by immunoblotting that full-length ∼100-kDa Jph2 was preferentially recognized. We do not know definitively whether a calpain cleaved COOH-terminal fragment would remain in the SR membrane associated with RyR2 clusters [or be translocated to the nucleus (55)] and be recognized by this Jph2 antibody in STORM images. The 2-D STORM images focusing on the distribution of Jph2 with RyR2 clusters showed 25% fewer RyR2 clusters per cell area and significantly fewer Jph2 localizations associated with those clusters in PTU cardiomyocytes compared with EU and T3-treated cells. As reported by other studies of diseased hearts, decreased Jph2 could result in loss of structured dyads, and subsequent disarray of T-tubules, similar to that shown here in TH-deficient cells. These adverse dyad changes might be expected to increase Ca^2+^ leak from SR stores leading to asynchronous calcium release and diminished contractility. Remarkably, the reorganization of TT-jSR dyads and RyR2 clusters to the *z*-lines with T3 treatment reflects a relatively rapid response to normalize cell ultrastructure. Whether this T3 effect is driven solely or partially by T3 upregulation of Jph2 is unclear, but data from animal studies either overexpressing Jph2 or reexpressing Jph2 in heart failure models support Jph2’s role in promoting ion channel organization, possibly driven by direct physical interactions of Jph2 with both RyR2 and LTCC (27, 28, 46). Many of the T3-regulated protein-coding genes and noncoding RNAs are necessary for the organization of ion channels, myofibrillar proteins, and organelles for optimal contractile function (2, 30, 48). Therefore, we contend that treatment of chronic heart diseases with T3 to normalize thyroid activity may have a greater impact on improving overall cardiac function than treatments targeting single molecules or gene therapy approaches aimed to deliver Jph2 or SR calcium ATPase (SERCA2a).

### Three-Dimensional STORM Imaging of Dyadic Proteins

We observed that in normal cells the average RyR2 cluster size was 221 nm (range was 190–245 nm) and 268 nm (204–309 nm) in 2-D and 3-D images, respectively; however, the average number of RyR2 localizations per cluster was less in 3-D than 2-D images (44 ± 8 vs. 69 ± 25, respectively). These results were not unexpected, assuming an overcount of superimposed blinking events in 2-D images that would otherwise be discerned along the axial *z*-plane in 3-D images (43). The total number of RyR2 localizations per cell area (ROI) in PTU cells and the percentage of these RyR2 localizations that were clustered was significantly less in PTU cells compared with either EU or PTU + T3 cardiomyocytes, suggestive of RyR2 tetramers located outside of dyads or extradyadic. Similar observations for RyR2 redistribution have been described in cardiomyocytes isolated from animal and human failing hearts (26, 28), which may be the result in part of the effects of decreased cellular T3 activity in those hearts (56–58). Current conjecture suggests that cluster size, volume, and RyR2 channel arrangements within clusters are key elements determining channel function (reviewed in Refs. 51 and 59). Herein, the 3-D image analysis showed that cluster sizes and volumes were similar among study groups, and with no significant differences in a number of RyR2 localizations per cluster, we can infer that the channel packing density did not change with thyroid condition.

Clusters that contained four to nine RyR2 localizations made up ∼30% of all clusters, and although the number of these smaller clusters was highly variable among cells and treatment groups, 20%–30% had no associated Jph2 or Ca_V_1.2. Notably, the distances between small clusters without Jph2 or Ca_V_1.2 and their nearest neighbor clusters were much greater than small clusters with colocalized Jph2 or Ca_V_1.2. Whether these small RyR2 clusters resided outside the TT-jSR dyad structures at sarcomere *z*-lines, and possibly in locations described as corbular SR could not be determined using the current imaging techniques (26). Recent imaging studies have shown that RyR2 distribution within clusters is a dynamic process influenced by ancillary proteins and covalent modifications and that clusters can fragment into smaller groupings as first reported in myocytes from failing hearts (42, 60). Sheard et al. (61) used enhanced expansion microscopy (EExM) and 3-D imaging to document “fraying” or fragmentation of RyR2 clusters with accompanying redistribution of Jph2 in myocytes from failing rat hearts. Whether the smaller clusters identified in the present study represent fragmentation of larger clusters will require these alternative nanoscale imaging approaches.

The question these observations raised was whether the significant reduction of total Jph2 per cell and decreased number of Jph2 localizations associated with each RyR2 cluster (and with fewer total clusters) was responsible for the pathological changes observed in dyad organization with loss of T-tubule integrity in thyroid deficient cardiomyocytes. One potential outcome of fewer Jph2 proteins within disrupted dyad structures of the PTU myocytes could be a loss of Ca_V_1.2 associated with RyR2 clusters. 3-D images showed a reduction of total Ca_V_1.2 per cell area (ROI) and per cluster and with significantly more clusters without any associated Ca_V_1.2 or without any Jph2. T3 treatment normalized the number of Jph2 localizations associated with RyR2 clusters; however, the number of Ca_V_1.2 localizations per cluster was unaffected by T3 and remained significantly lower than normal. Evidence of T3 regulation of transcription of the LTCC gene (specifically the channel’s pore forming α_1_-subunit, Cacna1c) is lacking although several studies have shown altered Cacna1c mRNA in response to T3, while other studies have focused on electrophysiological responses to T3 including Ca^2+^ current (*I*_CaL_) (62, 63). In contrast, TH effects on Ca^2+^ uptake by SR-Ca^2+ ^ATPase (SERCA2a) during diastole and its regulation by phospholamban (PLN) have been well studied (64, 65). Several decades of research have generated data supportive of thyroid hormone effects on cardiac ion channel activities, whether through genomic mechanisms or direct channel interactions (1, 2). Regardless, the organization of LTCC and RyR2 at dyad structures is crucial for synchronized Ca^2+^ release, and Gross et al. (28) have recently shown that direct interaction of Jph2 with LTCC is critical for dyad stabilization and effective EC coupling. In failing human cardiomyocytes, Hong et al. (66) have shown that LTCC redistributes away from T-tubules to the surface membrane resulting in reduced *I*_CaL_. These authors also showed that the scaffolding protein, BIN1 (bridging integrator1), which translocates Ca_V_1.2 to T-tubules was decreased. We have not imaged BIN1 but have shown previously that T3 had no effect on BIN1 mRNA expression in cardiomyocytes (30). Our present study results showing decreased Jph2 and Ca_V_1.2 associated with RyR2 clusters in thyroid deficient myocytes are supported by these other studies; however, T3 treatment did not increase Ca_V_1.2 while increasing Jph2 in clusters indicating the two responses appear independent. Our analysis compared mean values of all cell data, and comparing image data with immunolabeled Jph2 and Ca_V_1.2 in each cell would provide a better understanding of the relationship between Jph2 and Ca_V_1.2 in response to T3. Nevertheless, the observation that many pathological changes in PTU myocytes were largely reversed relatively rapidly by T3 treatment alone, supports its regulatory role in ion channel organization within the dyads that could ultimately affect EC coupling.

### Cardiomyocyte SR Ca^2+^ Sparks

In prior work, we recorded significant decreases in the contractile displacement curves and reduced velocities of Ca^2+^ transients in response to field stimulation of thyroid-deficient cardiomyocytes compared with either normal or T3-treated animals (30). Herein we imaged quiescent (not paced) Fluo4-loaded cardiomyocytes to record spontaneous Ca^2+^ sparks as a measure of SR calcium leak potentially triggered by altered RyR2 gating activity and/or organization in clusters. Ca^2+^ spark frequency and amplitude were significantly increased in PTU-derived cardiomyocytes, and the steepness of the spark upstroke and shorter temporal duration suggested localized, rapid Ca^2+^ release events. In normal and T3-treated myocytes, the duration (FDHM) of sparks was greater and the amplitude was lower compared with sparks in PTU cells. These observations may reflect changes in functional groupings of RyR2 clusters that produce Ca^2+ ^sparks (Ca^2+ ^release units, CRU) such as dispersion into smaller clusters as has been shown in failing cardiomyocytes (60). Furthermore, earlier studies have shown altered ion channel function in response to thyroid status including reduced SR Ca^2+^ content in hypothyroid cardiomyocytes (64). Recently, Hou et al. (67) recorded Ca^2+^ sparks in two spatial dimensions, and described two distinct types of Ca^2+^ spark events, single- and multirelease sparks with the latter exhibiting several temporally separated releases resulting in longer-lasting and larger-magnitude Ca^2+^ release. These authors illustrated how two-dimensional imaging produced profiles of multirelease sparks that could be misidentified using one-dimensional line-scan imaging that depends on line position, similar to that used in the present study. Despite these methodological limitations, our imaging results are best explained by T-tubule disarray seen with loss of thyroid function, which can lead to dyadic ion channel disorganization, impaired Ca^2+^ homeostasis, and SR Ca^2+^ leak that contribute to desynchronized EC coupling and reduced cardiac contractility.

### Study Limitations

A strength of this study is that the 2-D and 3-D image analyses produced supporting results, thus strengthening our conclusions. Current limitations are as follows: *1*) although we used fluorescent dye-conjugated primary antibodies against RyR2 proteins, we used fluorescent labeled-secondary antibodies targeting anti-Ca_V_1.2 and Jph2 primary antibodies; the superiority of DNA-PAINT technology in the resolution of proteins within <30 nm may improve quantitation of RyR2 tetramers; *2*) our studies would benefit from three color imaging with immunolabeled primary antibodies targeting each of the three proteins: RyR2, Jph2, and Ca_V_1.2; *3*) the length of T3 treatment used in this animal model was based on our prior studies; however, it would be of interest to document the earliest responses to treatment; and *4*) thyroid hormones elicit responses from all cell types, having effects on every organ system; therefore, the organismic changes associated with thyroid hormone deficiency including changes in systemic vasculature, blood volumes, cardiac myocyte growth, blood pressure affecting pre- and afterload, metabolism, neural, and immune activation, are all likely to have indirect effects on cardiac ultrastructure remodeling and ion channel organization studied herein. The current study outcomes support the clinical significance of monitoring and maintaining normal thyroid hormone function under all disease conditions due to its central regulatory role in physiological processes.

Our rationale focusing these studies on adult female rats was twofold: *1*) body weights of female rats plateau after ∼12 wk contrary to males, thereby facilitating studies of intervention-mediated cardiac growth and remodeling independently of body mass, and *2*) of clinical relevance is the higher risk of thyroid disease in females than males. This being said, the manifestations of thyroid dysfunction in heart disease may differ between the sexes, and the numerous observational studies examining thyroid dysfunction in human HF or in investigational studies using TH treatment have rarely considered sex as an independent contributing factor on outcomes (14, 68). A recent publication reported significant sex differences in normal mouse hearts in the organization of transverse and longitudinal tubules, and in RyR2 cluster size and density, with higher Ca^2+^ spark frequencies in females than males, but with similar Ca^2+ ^transients between sexes (69).

## DATA AVAILABILITY

The data generated or analyzed for this study are available from the corresponding authors upon reasonable request.

## GRANTS

This research was supported by National Heart, Lung, and Blood Institute Grant 1R15-HL-154068-01 (to K. M. Ojamaa).

## DISCLOSURES

No conflicts of interest, financial or otherwise, are declared by the authors.

## AUTHOR CONTRIBUTIONS

K.O., A.M.G., and R.F.S. conceived and designed research; A.C., N.N., S.S., S.M., A.T., D.S., J.F., and K.O. performed experiments; A.C., N.N., S.S., X.H., C.P.S., R.F.S., and K.O. analyzed data; A.C., N.N., S.S., A.M.G., R.F.S., and K.O. interpreted results of experiments; A.C., R.F.S., and K.O. prepared figures; A.C. and K.O. drafted manuscript; A.C., N.N., S.S., S.M., A.T., D.S., J.F., A.M.G., R.F.S., and K.O. edited and revised manuscript; A.C., N.N., S.S., S.M., A.T., D.S., J.F., X.H., C.P.S., A.M.G., R.F.S., and K.O. approved final version of manuscript.
